# Non-Operative vs. Operative Treatment of Pediatric Proximal Humerus Fractures: Surgery Offers No Clinical or Economic Benefit, a Retrospective Study of 152 Children

**DOI:** 10.3390/children13010067

**Published:** 2025-12-31

**Authors:** Tosca Cerasoli, Marina Magnani, Marco Todisco, Marianna Viotto, Grazia Chiara Menozzi, Giulia Alessandri, Cosma Caterina Guerra, Tiziana Pianta, Giulio Maria Marcheggiani Muccioli, Gino Rocca, Giovanni Trisolino

**Affiliations:** 1 II Orthopaedic and Traumatologic Clinic, IRCCS Istituto Ortopedico Rizzoli, 40136 Bologna, Italy; tosca.cerasoli@ior.it (T.C.); giuliomaria.marcheggianimuccioli@ior.it (G.M.M.M.); 2 Unit of Pediatric Orthopedics and Traumatology, IRCCS Istituto Ortopedico Rizzoli, 40136 Bologna, Italy; marina.magnani@ior.it (M.M.); marco.todisco@ior.it (M.T.); marianna.viotto@ior.it (M.V.); graziachiara.menozzi@ior.it (G.C.M.); giulia.alessandri@ior.it (G.A.); cosmacaterina.guerra@ior.it (C.C.G.); gino.rocca@ior.it (G.R.); 3 Unit of Programming, Control and Evaluation Systems, IRCCS Istituto Ortopedico Rizzoli, 40136 Bologna, Italy; tiziana.pianta@ior.it

**Keywords:** pediatric proximal humerus fracture, Neer–Horowitz classification, conservative treatment, non-operative management, surgical treatment, functional outcomes, QuickDASH, return to sport, remodeling, pediatric orthopedics

## Abstract

**Highlights:**

**What are the main findings?**

- Non-operative treatment of pediatric proximal humerus fractures provides excellent long-term functional outcomes, even in Neer–Horowitz grade III–IV injuries.
- Surgical management does not improve recovery, shoulder function, or return-to-sport rates compared with conservative care.

**What are the implications of the main findings?**

- Conservative treatment should remain the standard of care, with surgery reserved only for exceptional indications such as open fractures or neurovascular compromise.
- Avoiding unnecessary surgery reduces healthcare costs, minimizes postoperative issues, and limits the psychosocial burden for children and families.

**Abstract:**

Background: Pediatric proximal humerus fractures (PHFs) typically heal well due to their strong remodeling potential, supporting non-operative management even in displaced injuries. However, surgery for Neer–Horowitz grade III–IV fractures has become more frequent despite limited evidence of superior outcomes. Methods: A retrospective analysis of 152 children (<14 years) treated for isolated PHFs at a tertiary pediatric orthopedic center (2004–2023) was performed. Clinical records and telephone follow-up provided demographic data, fracture classification, management, complications, and functional outcomes (QuickDASH, Tegner, return to sport). A direct cost analysis compared conservative and surgical pathways. Results: Of 152 patients, 133 were treated non-operatively and 19 surgically. Conservative management achieved excellent results across all fracture types: nearly all patients reported normal QuickDASH scores and full shoulder function. Among Neer III–IV fractures (*n* = 37), functional outcomes, activity levels, and sport resumption were similar between treatment strategies. Minor transient issues (pin migration, temporary stiffness, delayed return to sport) occurred only after surgery. No meaningful complications were observed in the conservative cohort. Mean costs differed substantially: €1452.09 for non-operative management versus €7832.12 for surgical treatment. Conclusions: Long-term outcomes of pediatric PHFs were uniformly excellent, regardless of fracture severity or treatment modality. Surgery did not improve recovery, function, or return to sport and was associated with higher costs and minor postoperative issues. Conservative management should remain the standard of care for nearly all pediatric PHFs, with surgery reserved for exceptional circumstances such as open fractures, neurovascular compromise, or failed closed reduction.

## 1. Introduction

Proximal humerus fractures (PHFs) are relatively uncommon injuries in children, representing only 2–5% of all pediatric fractures [1]. The estimated incidence ranges from 31 to 680 per 100,000 children per year, with a clear male predominance and a peak incidence during early adolescence [2]. These fractures usually result from falls on an outstretched arm, direct trauma, or sports-related injuries, while motor vehicle accidents account for a smaller but significant proportion of cases [3].

The clinical importance of PHFs lies in the unique biology of the proximal humerus. The physis contributes up to 80% of humeral longitudinal growth, allowing substantial correction of deformity through remodeling, even in cases with marked displacement [1,2,3]. Combined with the wide range of motion (ROM) of the glenohumeral joint, this remodeling capacity means that most fractures, particularly those with minimal displacement (Neer–Horowitz grades I–II), heal uneventfully and with excellent functional outcomes when treated non-operatively [2,3]. Historical evidence already reflected this principle: in 1965, Neer stated that open treatment of PHFs in children was not justified, even in severely displaced injuries [4].

Nevertheless, debate persists regarding the optimal management of severely displaced fractures in older children and adolescents. Several systematic reviews and cohort studies have identified age at injury and degree of displacement as potential predictors of poorer outcomes [2,4,5]. For this reason, operative treatment has increasingly been considered for adolescents approaching skeletal maturity or for Neer–Horowitz grade III and IV fractures [3,6,7]. Common techniques include percutaneous pinning, elastic stable intramedullary nailing, and, less frequently, open reduction and internal fixation [4,7].

However, accumulating evidence suggests that surgery does not consistently provide superior results compared to conservative care. Functional outcomes, patient-reported quality of life, and return to sport are generally comparable between operative and non-operative cohorts [1,2,5,6]. Moreover, surgical intervention exposes children to anesthesia-related risks, potential perioperative complications, and longer hospitalization, while also increasing healthcare costs [1,2,6]. Recent meta-analyses have shown that the rate of operative treatment has risen to nearly one-third of all PHFs, with up to 60% of severely displaced fractures managed surgically, yet without convincing data supporting a corresponding benefit in postoperative function, complication rates, or return to sport [4].

These considerations support the notion that non-operative treatment remains the gold standard for the vast majority of pediatric PHFs, while surgery should be reserved for selected cases such as open fractures, neurovascular compromise, or failed closed reduction attempts.

This study investigates a large cohort of pediatric patients with proximal humerus fractures (Neer–Horowitz I–IV) with long-term follow-up, including functional assessment via QuickDASH, Tegner Activity Scale, and return-to-sport evaluation. The primary aim is to compare long-term clinical and functional outcomes, as well as direct healthcare costs, between operative and non-operative treatment. In particular, we asked whether surgical intervention for Neer III–IV fractures provides a meaningful advantage in terms of risk–benefit and cost-effectiveness, helping to guide evidence-based treatment decisions.

## 2. Materials and Methods

### 2.1. Study Design

We conducted a retrospective cohort study at the IRCCS Istituto Ortopedico Rizzoli, a tertiary referral hospital with a dedicated pediatric orthopedic unit. We included all consecutive pediatric patients (<14 years) presenting to the emergency department between 1 January 2004 and 1 January 2023 with an isolated closed proximal humeral fracture and open physes, provided they had at least one follow-up visit at our institution (minimum 90 days). Patients were excluded if they had pathological or open fractures, shoulder dislocation, neurovascular involvement, concomitant fractures at other sites, skeletal maturity, or if radiographic documentation or clinical records at baseline were incomplete. All cases were managed within the pediatric orthopedic unit following emergency department admission. Parents or legal guardians provided written informed consent for the use of personal data for research purposes. The study was conducted in accordance with the principles of the Declaration of Helsinki and reported following the STROBE guidelines for observational studies.

After approval from the local ethics committee (protocol code CE-AVEC 827/2019/Oss/IOR), emergency department registries were systematically screened. Potentially eligible patients were verified by 3 independent observers (M.M., M.V., M.T.) checking the radiographic and clinical records to confirm inclusion criteria. Only those fulfilling all requirements and with explicit consent from patients or guardians were enrolled.

### 2.2. Patient Assessment

For each case, demographic characteristics, comorbidities, fracture classification, treatment modality (non-operative vs. operative, percutaneous vs. open), perioperative variables, and follow-up outcomes were extracted from the institutional electronic medical records and through a telephone interview (QuickDASH, Tegner score and RTS). Radiographic images were independently reviewed by two authors, and fracture morphology consistently classified according to Neer’s pediatric classification. Greenstick and torus patterns were standardized under Neer type I. In cases of disagreement between the two primary reviewers, a third independent author was consulted to reach consensus and ensure methodological rigor.

Follow-up data were collected through scheduled outpatient visits and structured telephone interviews. All patients were contacted for telephone follow-up. In patients reachable by phone, return-to-sport and functional outcomes were assessed using validated instruments (Italian QuickDASH and Tegner Activity Scale). For patients not reachable by telephone, follow-up duration, return-to-sport status, and functional outcomes were extracted from the most recent clinical follow-up visits and corresponding medical records.

During the telephone interview, parents or patients (if of legal age) reported current symptoms, perceived shoulder function, and residual limitations.

Functional status was assessed using the Italian QuickDASH [8] administered verbally. The QuickDASH includes 11 items and a 4-item sports/performance module, is validated for children ≥8 years [9], and yields scores from 0 (no disability) to 100 (maximal disability).

Because baseline values were unavailable, results were compared with normative data for young adults, and the work module was omitted due to patient age. QuickDASH scores ≤7 and sports-module scores ≤6 in males and ≤13 in females fall within the first quartile of healthy controls, indicating no perceived upper-limb impairment [10]. This approach has been used in similar studies lacking baseline PROMs [11,12].

Detailed information on sports participation (type of activity and weekly frequency) was collected, and the Tegner Activity Scale was calculated accordingly. At the end of the interview, all respondents were offered the possibility of an in-person clinical evaluation; however, none of the patients or guardians deemed an additional visit necessary.

### 2.3. Cost Analysis

For patients undergoing surgical treatment, cost analysis included pre-admission expenses, inpatient stay costs (calculated using actual length of stay multiplied by the standard pediatric daily rate), and operating room costs, encompassing personnel, diagnostics, medical devices, and fixed overheads (e.g., maintenance, utilities, sterilization), based on the actual duration of surgery. Costs of five post-discharge follow-up visits at 7 and 30 days and at 3, 6, and 9 months were also included, covering personnel and imaging (one shoulder/upper-limb radiograph per visit).

For patients managed non-operatively, costs included emergency department access (assumed duration of 2 h, including personnel, medical devices, imaging, and fixed hourly ED costs), as well as the same five post-discharge follow-up visits with associated personnel and imaging costs.

### 2.4. Statistical Analysis

Continuous variables were summarized as mean ± standard deviation (SD); categorical variables as counts and percentages. Missing data were not imputed, and analyses were performed on available cases only. Preliminary data review showed that all surgically treated fractures had greater displacement (Neer type > II); therefore, subgroup analyses were performed to identify non-operatively treated patients with comparable fracture severity and displacement for meaningful comparison with the surgical group. Group comparisons of continuous outcomes (e.g., operative time, age, follow-up, QuickDASH, Tegner score) used Welch’s *t* test (unequal variances); Mann–Whitney U tests were run as non-parametric sensitivity analyses. Associations between ordinal Neer fracture type and age, Tegner score and QuickDASH were examined with Spearman’s rank correlation and, where appropriate, Kruskal–Wallis tests. Relationships between categorical variables (e.g., sex vs. operation status; technique [open vs. percutaneous] vs. type) used Fisher’s exact test or χ^2^ tests as appropriate. Two-sided 95% confidence intervals were calculated for effect estimates, and statistical significance was defined as α = 0.05, and *p* value < 0.05, without adjustment for multiple testing given the exploratory nature and small subgroup sizes. Fracture morphology terms were standardized prior to analysis (e.g., greenstick and torus were coded under Neer type I). All analyses were conducted in Python (SciPy 3.10 version).

## 3. Results

**Figure 1:**
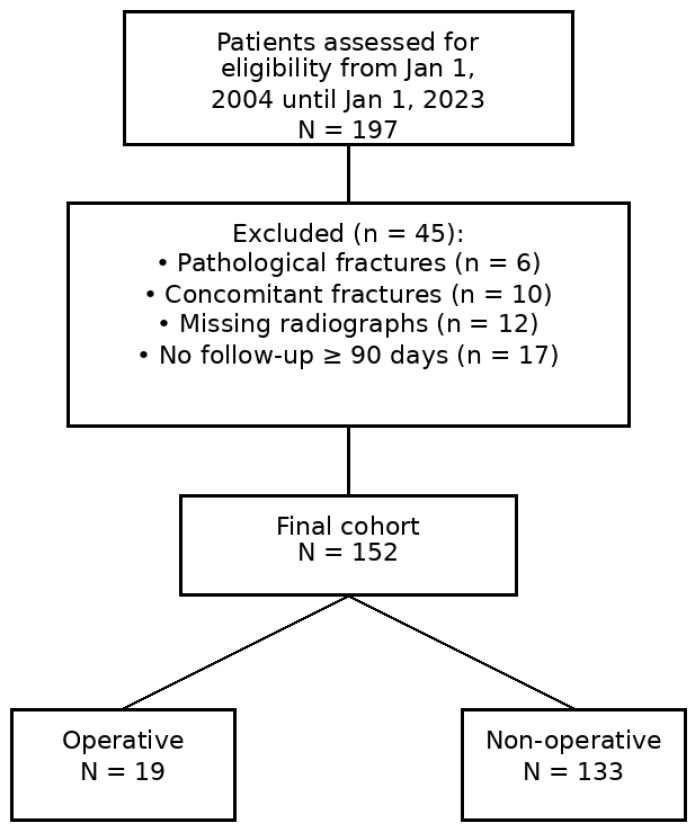
STROBE flow-chart for retrospective cohort studies.

We analyzed 152 children, 133 managed non-operatively and 19 surgically (Figure 1). A total of 48 patients could not be reached by telephone for follow-up reassessment (43 of the non-operative group, 5 of the operative group) (Tables S1 and S2).

### 3.1. Non-Operative Group

Among the 133 patients treated non-operatively, there were 62 type I Neer fractures (including greenstick *n* = 12 and torus *n* = 14), 53 type II, 16 type III, and 2 type IV. All patients managed conservatively were treated with a standard arm sling, except for four cases that underwent closed reduction under anesthesia and were subsequently immobilized with a Velpeau bandage for 30 days.

The mean follow-up was 59.7 ± 47.1 months (95% CI, 51.7–67.7). No statistical differences among follow-up were detected (H = 4.47, *p* = 0.215). The sex distribution was 67 males (M) and 66 females (F). Comorbidities were present in 10 patients: petit-mal epilepsy (*n* = 2), scoliosis under conservative treatment (*n* = 6), prior calcaneo-stop for flatfoot (*n* = 1), and humeral diaphyseal fibroma incidentally identified during the diagnostic work-up for the fracture (*n* = 1).

**Figure 2:**
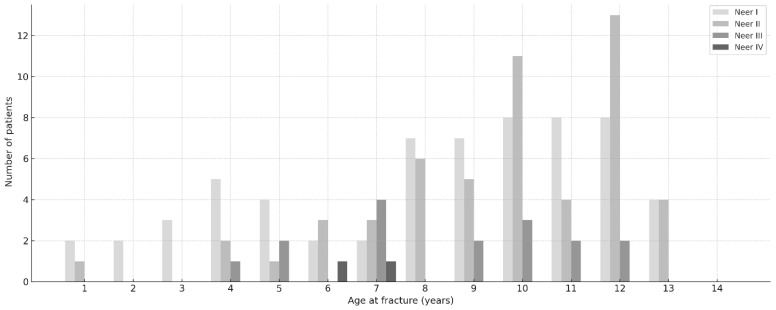
Age distribution by fracture. Histogram of the study cohort showing age at fracture (years) on the *x*-axis and number of patients on the *y*-axis.

The mean age at fracture was 8.9 ± 3.0 years, stratified by Neer type as follows: type I—8.3 ± 3.4 years (range 1–13 y), type II—9.9 ± 2.7 years (range 1–13 y), and type III—8.5 ± 2.6 years (range 4–12 y). For type IV, only two patients were included, with a mean age of 6.5 y (range 6–7 y). No association was detected between age and Neer type (Spearman ρ = 0.088, *p* = 0.320; Kruskal–Wallis H = 4.5, *p* = 0.104). The age distribution is depicted in Figure 2.

QuickDASH scores were available for 90 patients treated non-operatively. Of these, 89/90 patients (98.9%) reported an excellent QuickDASH score (≤7 points), and 85/90 (94.4%) achieved the best possible score (QuickDASH = 0).

Only two minor events (1.4%) were observed, both in 13-year-old boys with Neer I fractures. One developed a brief skin irritation after sling removal, and the other reported temporary shoulder instability; both resolved completely. At a mean follow-up of 88 months, both children had a QuickDASH of 0 and were fully active in sport. No range-of-motion deficits were identified at last follow-up in any patients, and all children demonstrated full restoration of shoulder function.

**Figure 3:**
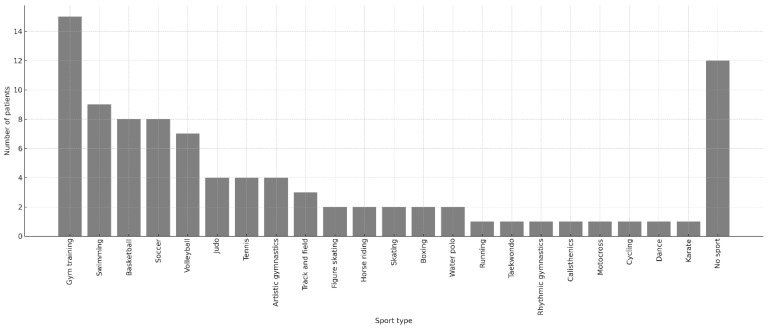
Bar chart illustrating the number of patients participating in each sport following proximal humerus fracture. Each sport practiced was counted as a separate entry; therefore, children who practiced two sports contributed two independent data points. A total of 80 sport entries were recorded. The “No sport” category includes patients who reported not engaging in any physical activity (*n* = 12). Patients who were unreachable for telephone follow-up (*n* = 43) were excluded from this figure to improve visualization of individual sport frequencies.

RTS information was available for 92 children (90 obtained through telephone interviews and 2 from outpatient follow-up records; see Figure 3). Twelve patients reported no participation in sport. Among those who returned to sport, the most frequently practiced activities were gym-based exercise (*n* = 15), swimming (*n* = 9), basketball (*n* = 8), soccer (*n* = 8), and volleyball (*n* = 7). Less common activities included martial arts, racket sports, artistic gymnastics, athletics, water polo, skating disciplines, and various individual sports.

The post-injury Tegner score was 6.7 ± 1.7 (IC 95% 6.5–7.2). Activity levels were comparable across all Neer fracture groups, with no statistically significant differences detected (*p* = 0.32).

**Figure 4:**
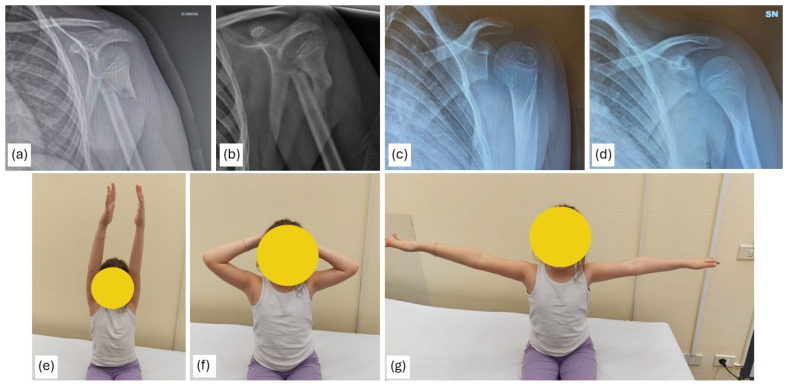
A 7-year-old patient with a Neer type IV fracture treated conservatively. Follow-up radiographs at 1 month (**a**,**b**) and at 10 months are shown (**c**,**d**). Clinical follow-up at 10 months demonstrated complete restoration of joint range of motion (**e**–**g**).

An example of a Neer type IV fracture treated non-operatively is shown in Figure 4.

### 3.2. Cost Analysis in Non-Operative Group

The initial Emergency Department (ED) evaluation cost €477.48, comprising standard 2 h resource utilization (€160.49), proximal humerus X-rays (€106.80), orthopedic physician time (€140.00), nursing staff (€52.00), and the immobilization device (€18.19).

While most patients received a simple shoulder immobilization brace, a subset was treated with a Desault/Velpeau-type sling, which carries comparable material costs.

A small proportion of children (*n* = 4) presented with acute traumatic deformity requiring closed reduction under anesthesia, followed by Velpeau bandaging. For these cases, the cost model also includes the anesthesiologist fee, calculated at €70/h, increasing the total ED cost accordingly.

Post-ED follow-up accounted for an additional €974.61, with each scheduled visit costing €194.92 (orthopedic physician €35.00, nursing staff €13.00, radiographic imaging €106.80, and standard space utilization €40.12). This structure reflects routine checks at 7 days, 30 days, and at 3, 6, and 9 months.

In clinical practice, follow-up duration varied considerably: some patients required additional long-term annual evaluations, whereas others discontinued follow-up after the 3-month visit once complete clinical recovery was documented.

Overall, considering the ED encounter, immobilization needs, potential reductions under anesthesia, and the typical pattern of subsequent follow-up, the estimated mean cost for non-operative management is €1452.09 per patient, with higher costs expected in cases requiring anesthetic reduction.

### 3.3. Operative Group

Among the 19 surgically treated patients 13 were classified as Neer III fractures and 6 as Neer IV fractures.

The mean follow-up was 89.9 ± 47.4 months. The difference in follow-up duration between Neer III and Neer IV groups was slightly statistically significant (Welch’s *t*-test *p* = 0.044; Mann–Whitney *p* = 0.036).

**Figure 5:**
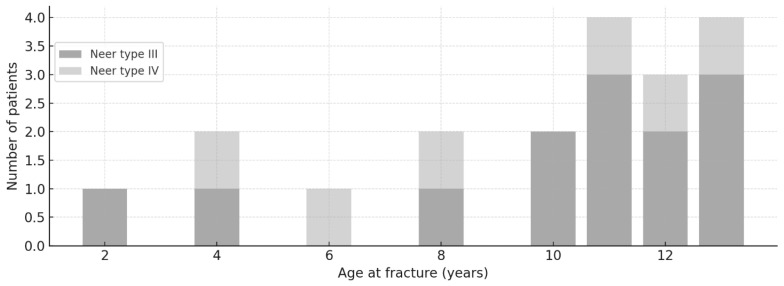
Neer fracture type by age. Stacked bar chart showing the number of patients (*y*-axis) at each year of age at fracture (*x*-axis). Bars are grouped by Neer type (III and IV).

The mean age at fracture was 9.7 ± 3.4 years (as depicted in Figure 5); no statistically significant difference was detected between age at fracture in type III and IV.

The sex distribution was 6 males and 13 females; no statistically significant difference in fracture type distribution according to patient sex was detected.

Among the fourteen patients who completed the telephone follow-up, functional limitations were exceedingly rare: only two children reported a QuickDASH value different from zero, both scoring 2.3. One of these was a Neer III fracture treated surgically, with the patient fully engaged in basketball at 44 months of follow-up. The other was a female patient with a Neer IV fracture, who reported a similarly minimal score while actively practicing swimming after 101 months of follow-up. All remaining respondents had a QuickDASH of 0.

**Figure 6:**
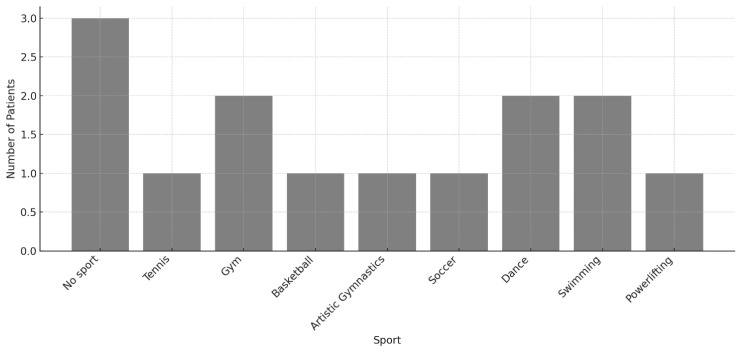
Return-to-sport distribution in the study cohort. When combining all operative patients with available follow-up (*n* = 14), the most frequent sport categories were gym/fitness (*n* = 2), dance (*n* = 2), and swimming (*n* = 2). Powerlifting, tennis, basketball, artistic gymnastics, and soccer were each reported by one patient.

RTS information was available for 14 of the 19 surgically treated patients. Among Neer III fractures, 6 of 8 respondents returned to sport, while 2 reported no interest in participating in sports. All six Neer IV patients provided RTS data, with four resuming sport and one remaining inactive. The distribution of sport activities is illustrated in Figure 6.

The overall mean Tegner score at final follow-up was 6.5 ± 1.9 (95% CI, 5.4–7.7). Stratification by fracture type showed comparable functional recovery between groups (Welch’s *t*-test *p* = 0.18; Mann–Whitney *p* = 0.29), indicating similar return-to-activity levels regardless of fracture severity.

By fracture type, five Neer III fractures were managed with an open approach, six Neer III fractures were managed percutaneously, and for two Neer III fractures the technique was unspecified, whereas Neer IV fractures were treated with four open and two percutaneous procedures. The overall mean operative time was 49.8 ± 20.4 min. Open procedures lasted 64.0 ± 10.1 min versus 33.8 ± 16.9 min for percutaneous procedures. A statistically significant difference among approaches was detected (mean difference 30.3 min; 95% CI 15.2–45.3; Welch *p* = 0.001; Mann–Whitney *p* = 0.002). Operative time did not differ by Neer type (type III 45.4 ± 23.6 vs. type IV 57.8 ± 10.2 min; Spearman ρ = 0.327, *p* = 0.200; Mann–Whitney *p* = 0.209).

Percutaneous pins were routinely removed in the outpatient clinic at 30 days. Only one child had internal implants; these became symptomatic and required removal in the operating room. The mean hospital stay was 4.5 ± 1.5 days.

Minor complications occurred in a small proportion of patients. One child experienced early pin migration at 14 days, managed non-operatively with Velpeau immobilization; full shoulder function and a QuickDASH of 0 were documented at long-term follow-up. Delayed recovery of shoulder motion was noted in three patients (15.7%). Two children showed a mild (10°) internal-rotation deficit at 3 months that fully resolved over time, and both returned to their usual sport activities with normal function. Another child treated with an open approach experienced more prolonged stiffness and pain, delaying return to sport, but ultimately achieved complete recovery, full ROM, and a QuickDASH of 0.

**Figure 7:**
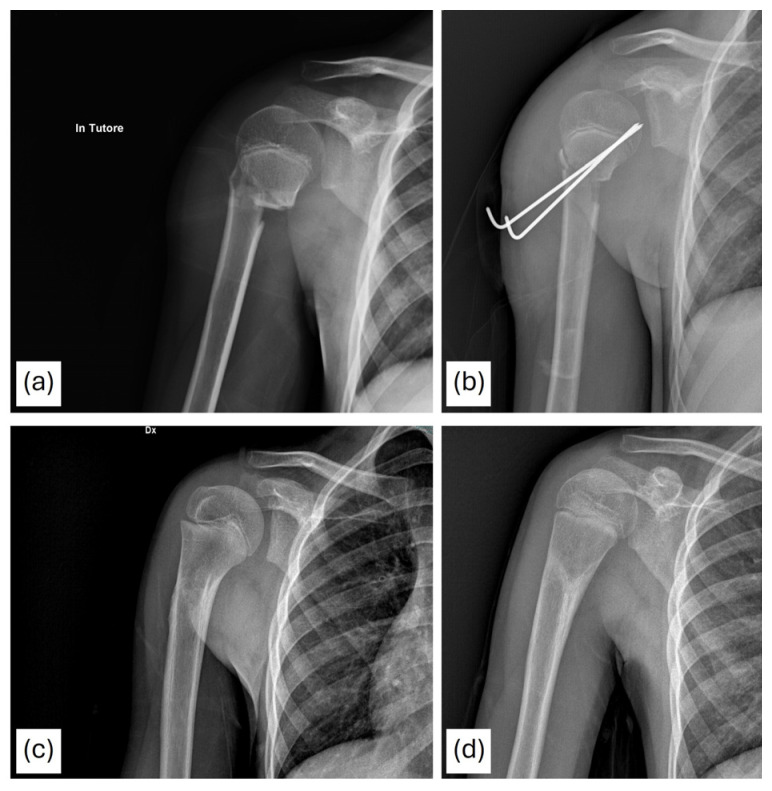
A 10-year-old patient with a Neer type IV fracture treated surgically by closed reduction and fixation with Kirschner wires. Postoperative radiographs are presented (**a**,**b**), along with a radiographic follow-up at 6 months (**c**,**d**).

An example of a fracture treated with percutaneous pinning is shown in Figure 7.

### 3.4. Cost Analysis in Operative Group

For surgically treated patients, total inpatient costs included hospitalization, operating room resources, preoperative assessment, and scheduled postoperative visits. The mean cost per surgical case was €7832.12 (range €5367.06–€12,905.06), compared with an average reimbursement of €3366.64 from the regional healthcare system.

Hospitalization represented the largest cost component. Each inpatient day cost €1101.94 (including general and administrative overhead), resulting in a total ward cost ranging from €3305.81 to €9917.44 depending on length of stay (3–9 days). Operating room time added a further major expense: with an hourly rate of €891.65, total OR costs varied according to surgical duration, ranging from €505.52 to €2998.42.

All patients underwent a pre-operative evaluation including anesthesiology review and radiographic imaging, at a fixed cost of €229.92. Post-operative management required routine scheduled follow-up, with each visit costing €194.92 (orthopedic surgeon €35.00, nursing staff €13.00, radiographs €106.80, and facility usage €40.12), for a total follow-up cost of €974.60 per surgical patient.

A distinction by fracture type did not translate into a substantial economic difference. Neer III fractures and Neer IV fractures showed broadly comparable total costs, with overlapping ranges for hospitalization, operating room time, and follow-up.

### 3.5. Operative vs. Non-Operative Comparisons

A total of 37 patients presented with Neer III and IV proximal humerus fractures: 18 were treated non-operatively and 19 underwent surgery. The non-operative and operative groups were comparable at baseline, with similar age at injury (8.5 ± 2.6 vs. 9.7 ± 3.4 years) and a similar sex distribution. Follow-up duration was longer in the operative cohort (89.9 ± 47.4 vs. 47.7 ± 46.1 months) with a slightly statistical significant difference (Welch’s *t*-test *p* = 0.044; Mann–Whitney *p* = 0.036). A comparable proportion of patients did not respond to the telemedicine follow-up, 38.5% in the operative group and 31.2% in the non-operative cohort.

Functional outcomes were excellent in both groups. All non-operatively managed patients with available data reported a QuickDASH score of 0, whereas in the operative group two patients reported minimal residual symptoms (QuickDASH 2.3). Activity levels mirrored the same pattern: the mean Tegner score was 7.6 ± 0.8 (IC 95% 7.0–8.1) in the non-operative group and 6.1 ± 2.3 (IC 95% 4.1–8.2) in the operative group, indicating full recovery of pre-injury sport participation regardless of treatment strategy. No statistically significant differences were found between the two groups (Welch’s *t*-test *p* = 0.16; Mann–Whitney *p* = 0.16).

RTS rates were high and comparable, with 12 of 18 non-operative patients and 11 of 19 surgically treated patients resuming physical activity at follow-up, spanning a broad range of competitive and recreational disciplines many of which require substantial upper-limb engagement or repetitive overhead movements.

Severe complications were absent in both treatment groups, however minor postoperative issues were reported in the operative cohort, one early pin migration, two transient internal rotation deficits, one severe delayed return to sport, but were resolved completely at long-term follow-up. Cost analysis showed a marked difference between treatment strategies: non-operative management averaged €1452.09 per patient, whereas operative treatment averaged €7832.12, largely due to hospitalization and operating room costs, resulting in a mean loss of €4465.49 per surgical admission for the hospital. The results of all statistical comparisons performed are summarized in Table 1.

## 4. Discussion

In this large single-center cohort, non-operative treatment proved highly effective across fracture types, reflecting the strong remodeling potential of the pediatric proximal humerus [1,2,3]. Surgical management also resulted in good long-term outcomes, but it was associated with a greater number of minor postoperative events and substantially higher healthcare costs, without offering measurable functional advantages over conservative care.

More than 85% of fractures in our population were Neer–Horowitz type I or II, and all healed uneventfully with full restoration of shoulder motion and extremely low rates of functional abnormalities. QuickDASH scores were normal in 98.9% of cases, and no child demonstrated persistent range-of-motion limitations at long-term follow-up. Neer III fractures were less frequent (*n* = 29) and were treated either conservatively (*n* = 16) or surgically (*n* = 13). Long-term outcomes were similar between approaches, with full restoration of shoulder function, normal QuickDASH scores and comparable RTS levels, indicating that surgery did not provide measurable advantages for this fracture type. Neer IV injuries were rare (*n* = 8), and only two were managed non-operatively. Outcomes in these children were generally good and broadly comparable between operative and non-operative cases; however, the small sample size did not allow statistical comparison, two notable complications occurred: one early loss of fixation pins and one reoperation for implant removal.

These results reinforce the principle that non-operative care should remain the gold standard for most PHFs in children, moreover a substantial difference also emerged in economic terms: surgical management was approximately five times more expensive than conservative treatment, despite yielding comparable clinical outcomes.

Our findings align with previous reports demonstrating that pediatric PHFs are frequently minimally displaced and reliably remodel without surgical intervention [1,2]. Baker et al. reported that non-displaced or minimally displaced PHFs treated non-operatively had negligible risk of long-term complications, with no patient requiring secondary surgery [1]. Similarly, Abbot et al. found that children under 10 years of age generally achieve excellent functional outcomes irrespective of fracture grade, further supporting conservative management in younger patients [5]. Consistent with this evidence, during telephone follow-up many families reported that the child had completely forgotten about the injury, highlighting the absence of lingering symptoms or functional limitations years after the fracture.

The main controversy concerns severely displaced fractures in older children and adolescents, who have reduced remodeling potential. Some systematic reviews and meta-analyses suggest that increasing age and displacement are predictors of poorer outcomes [2,4,5]. As a result, operative fixation has become more frequent, with up to one-third of all PHFs now treated surgically [4]. In our cohort, all operated fractures were Neer type III or IV, reflecting this trend. These fractures occurred predominantly in children aged 10–13 years, although four younger patients (<6 years) were also treated surgically. Our results demonstrate that surgery does not confer measurable functional benefits compared with conservative treatment. Functional outcomes, RTS rates, Tegner activity levels, and long-term ROM were essentially identical between operative and non-operative groups. All Neer III and IV patients, regardless of treatment strategy, achieved full recovery, resumed sport activities involving high-demand upper-limb functions, and reported normal QuickDASH scores.

Importantly, true complications were absent in both groups. Minor transient issues, such as a temporary internal rotation deficit or delayed return to sport, occurred only among surgically treated patients and fully resolved over time. In contrast, complications related to fixation (early pin loss, reoperation for hardware removal) occurred exclusively in the operative group. These findings suggest that surgery introduces potential risks without offering superior outcomes.

Return-to-sport analysis further reinforces this conclusion. Children in both groups resumed a wide range of activities, many requiring repetitive overhead motion or substantial upper-limb engagement, such as basketball, volleyball, swimming, artistic gymnastics, judo, boxing, tennis, athletics, and dance, without functional limitations. Two surgically treated patients did not participate in sports at follow-up, but both had QuickDASH = 0, indicating that their inactivity reflected personal preference rather than shoulder impairment. Conversely, the only child with a minimal QuickDASH alteration (2.3) was fully active and regularly practicing basketball, demonstrating that subjective symptoms did not hinder sport participation.

Accordingly to our series the literature indicates that surgical intervention does not accelerate recovery [13]. Abbot et al. showed that functional and quality-of-life outcomes did not differ between operative and non-operative groups, while older children managed surgically still reported occasional residual symptoms [2]. Our results mirror these findings: time to return to sport was not shortened by surgery, and two patients developed transient deficits in internal rotation after operative treatment, whereas no deficits were observed in the conservatively treated cohort. Notably, within the surgical group, one child required as long as 40 months before returning to sport, and another needed 6 months to fully recover joint ROM.

Another critical consideration is the risk–benefit ratio. Bringing a child to the operating room exposes them to anesthesia and perioperative risks, while also increasing length of stay and costs for healthcare systems [1,2,6]. Earlier reports by Neer emphasized that pediatric proximal humerus fractures rarely required surgical treatment, given their remarkable capacity for remodeling [4]. Nevertheless, in the decades that followed, operative fixation became increasingly adopted, particularly for displaced fractures in older children and adolescents. Recent national data, however, suggest that this trend is now reversing, with surgical rates for pediatric PHFs declining in recent years, reflecting growing recognition that conservative treatment suffices in most cases [14].

The psychological burden of surgery must also be considered. Pediatric surgical hospitalization has been associated with heightened anxiety in both children and their families [15,16,17]. The fact that PHF surgery is an urgent, but not emergent, procedure often leaves little time for families to adapt or prepare, amplifying stress and distress. Evidence indicates that preoperative anxiety is closely linked to slower recovery, increased pain, and negative long-term psychological sequelae [16,17]. By avoiding surgery in cases where it is not strictly necessary, these negative psychosocial consequences can be minimized.

Economic aspects are equally relevant. Intraoperative management of PHFs, whether closed or open reduction, requires substantial use of imaging; at our institution, a mean of 20 intraoperative radiographs is obtained per case. This not only increases costs but also adds radiation exposure. In Canada, implementation of standardized care pathways has successfully reduced unnecessary radiographs and follow-up visits, yielding significant cost savings [18]. Moreover, surgical fixation has been shown to be considerably more expensive than conservative care, without demonstrable benefit in outcomes [19].

Taken together, our findings and the available evidence support a restrained approach to surgery. Conservative management ensures excellent results in the majority of cases, while avoiding unnecessary risks, psychological distress, and healthcare costs. Surgical treatment of pediatric PHFs should therefore remain exceptional, reserved for open fractures, neurovascular compromise, polytrauma, or failed attempts at closed reduction [20,21].

### Limitations

This retrospective study has inherent limitations, including potential selection bias and variable follow-up. Over recent years, we have progressively favored conservative treatment even for more severe fractures (Neer III–IV), based on both literature and growing clinical experience. All patients, past and recent, were contacted together, resulting in differences in follow-up duration. Neer IV fractures were almost exclusively treated surgically, limiting comparisons between operative and non-operative management. Importantly, the uniformly excellent QuickDASH and TAS outcomes at a mean follow-up of 48 months in conservatively treated patients support the notion that surgery does not accelerate—and may even delay—complete recovery.

Telephone follow-up was unavailable for nearly one-third of patients; while clinical records provided long-term information on complications, major functional limitations, and additional interventions in non-responders, patient-reported outcomes and return-to-sport details were largely missing. Nevertheless, the excellent clinical status and unrestricted return to overhead sports among responders, along with the absence of significant issues and overall good clinical–functional recovery in non-responders, suggest similarly favorable shoulder outcomes in this group.

QuickDASH normative values derived from young adult populations may not fully reflect age-specific function in children and adolescents (mean age ~14 years at assessment). Notably, 97% of patients demonstrated a ceiling effect, with only one patient scoring > 7. This likely reflects a combination of factors: the excellent healing and remodeling capacity of proximal humeral fractures in children, the long follow-up period reducing symptom recall, and known limitations of QuickDASH in pediatric populations, where ceiling effects have been reported even in more severe fractures with shorter follow-up [9]. Other studies have similarly documented high ceiling effects and excellent scores in pediatric upper-limb populations [22,23], suggesting limited sensitivity of these PROMs to detect subtle impairments in long-term follow-up. Whether the observed ceiling effect reflects true full recovery or reduced discriminative ability of QuickDASH remains unclear.

Importantly, all families were offered in-person clinical evaluation, but none opted for it, as all children were asymptomatic and functioning well. Although reliance on telephone follow-up is a limitation, the consistently good outcomes and absence of reported symptoms indicate that additional clinical assessments would unlikely have revealed further findings.

Finally, although surgical treatment is generally more costly than non-surgical management, our cost analysis reflects the Italian healthcare system and may not be directly generalizable to other settings.

## 5. Conclusions

In this large cohort of pediatric proximal humerus fractures, non-operative management resulted in excellent outcomes in nearly all cases, with minimal complications and full restoration of shoulder function. For Neer III and IV fractures, surgery did not provide faster recovery, superior function, or earlier return to sport. On the contrary, operative treatment entailed higher healthcare costs and occasional transient issues without clear clinical advantages. These findings, aligned with accumulating evidence, support conservative treatment as the standard of care for the vast majority of pediatric PHFs, reserving surgery for exceptional indications only. Limiting unnecessary surgical interventions optimizes recovery, minimizes risk and psychosocial burden, and reduces healthcare expenditures for children and their families.

## Figures and Tables

**Table 1:** Summary of statistical comparisons between demographic, clinical, and outcome variables according to fracture type and treatment strategy.

Comparison	Groups Compared	Variable Analyzed	*p*-Value
Age vs. Neer fracture type	Neer I–IV	Age at fracture	0.320; 0.104
Follow-up duration by Neer type (non-operative group)	Neer I vs. II vs. III vs. IV	Follow-up (months)	0.215
Age comparison (operative vs. non-operative, Neer III–IV only)	Operative vs. non-operative	Age at fracture (years)	>0.05
Sex distribution by treatment	Operative vs. non-operative	Sex (M/F)	>0.05
Follow-up duration (operative vs. non-operative)	Operative vs. non-operative	Follow-up (months)	0.044; 0.036
QuickDASH (operative vs. non-operative)	Operative vs. non-operative	QuickDASH score	0.16
Tegner score (operative vs. non-operative)	Operative vs. non-operative	Tegner score	0.16
Return to sport (RTS) rate	Operative vs. non-operative	RTS yes/no	>0.05
Age vs. Neer fracture type	Entire cohort	Age	0.320
Neer type vs. Tegner score	Entire cohort	Tegner score	>0.05
Neer type vs. QuickDASH	Entire cohort	QuickDASH	>0.05
Operative technique vs. operative time	Open vs. percutaneous	Operative time (min)	0.001; 0.002
Neer type vs. operative time	Neer III vs. Neer IV	Operative time	0.200; 0.209

## Data Availability

The raw data supporting the conclusions of this article will be made available by the authors on request.

## Supplementary Materials

The following supporting information can be downloaded at: https://www.mdpi.com/article/10.3390/children13010067/s1, Table S1: Demographic, clinical, and functional characteristics of patients treated surgically for proximal humeral fractures; Table S2: Demographic, clinical, and functional characteristics of patients treated conservatively for proximal humeral fractures.

## Abbreviations

The following abbreviations are used in this manuscript:

PHF	Proximal humerus fractures
ROM	Range of Motion
RTS	Return-to-Sport
SD	Standard deviation
ED	Emergency Department
