# Supplementary material for: Non-Operative vs. Operative Treatment of Pediatric Proximal Humerus Fractures: Surgery Offers No Clinical or Economic Benefit, a Retrospective Study of 152 Children
Source: Children (Basel). 2025 Dec 31;13(1):67. doi: 10.3390/children13010067 (PMC12840326; doi:10.3390/children13010067)
Supplement: Supplementary file 1 [file children-13-00067-s001.zip › children-4056921-supplementary.pdf]

Supplementary materials

Table S1: Demographic, clinical, and functional characteristics of patients treated surgically for proximal humeral fractures. Data include age at the time of fracture, follow-up duration, sex, Neer fracture classification, occurrence of complications, QuickDASH score, post-injury sport participation, and Tegner activity score. Missing data are reported as NA.

**Patient ID**	**Age at fracture (years)**	**Follow-up (months)**	**Sex**	**Neer fracture type**	**Complications**	**QuickDASH**	**Post-injury sport**	**Tegner score**
DEL	10.0	112	F	3	0	0	Gym	7
EY	8.0	146	F	3	0	0	No sport	3
FM	11.0	2	F	3	0	NA	NA	NA
BM	10.0	152	M	3	0	0	Tennis	8
BG5	12.0	5	F	3	0	NA	NA	NA
CN	13.0	1	F	3	0	NA	NA	NA
CN2	11.0	44	M	3	Delayed recovery	2.3	Basketball	8
EMY	2.0	1	M	3	0	NA	NA	NA
FB	4.0	72	F	3	0	0	Artistic gymnastics	8
NL	12.0	72	M	3	0	0	Soccer	8
RE	13.0	4	F	3	0	NA	NA	NA
RA	11.0	122	F	3	Delayed recovery	0	Dance	6
TM	13.0	96	F	3	0	0	No sport	3
DEV	13.0	88	F	4	0	0	Swimming	6
FS	6.0	113	F	4	Early pin migration	0	Dance	6
LA	11.0	155	M	4	0	0	Powerlifting	9
MM	4.0	101	F	4	0	2.3	Swimming	6
MS	8.0	136	M	4	0	0	Gym	7
OA	12.0	81	F	4	0	0	No sport	3

Table S2: Demographic, clinical, and functional characteristics of patients treated conservatively for proximal humeral fractures. Data include age at the time of fracture, follow-up duration, sex, Neer fracture classification, occurrence of complications, QuickDASH score, post-injury sport participation, and Tegner activity score. Missing data are reported as NA.

Patient ID	Age at fracture (years)	Follow-up (months)	Sex	Neer fracture type	Complications	QuickDASH	Post-injury sport	Tegner score
FAM	8,00	3	F	1	0	NA	NA	NA
FPG	11,00	65	M	1	0	0	Basketball	8
FG	8,00	68	M	1	0	0	Judo	8
AN	1,00	56	F	1	0	0	No sport	3
AA	10,00	68	M	1	0	0	Judo	8
AD	11,00	84	M	1	0	0	Gym	7
AA3	8,00	64	F	1	0	0	Swimming	6
BS	3,00	3	M	1	0	NA	NA	NA
BE2	5,00	179	M	1	0	0	No sport	3
BA	10,00	3	F	1	0	NA	NA	NA
BG	11,00	62	F	1	0	0	Swimming	6
BV	9,00	3	F	1	0	NA	NA	NA
BG2	9,00	3	F	1	0	NA	NA	NA
BM	10,00	82	F	1	0	0	Artistic gymnastics	8
CE	5,00	3	M	1	0	NA	NA	NA
CLK	10,00	3	M	1	0	NA	NA	NA
CG	10,00	102	F	1	0	0	Figure skating	8
CS	12,00	251	F	1	0	0	No sport	3
CF	12,00	3	M	1	0	NA	NA	NA
CB	12,00	74	F	1	0	0	Horse riding / tennis	7
CM	12,00	62	F	1	0	0	Taekwondo	8
CA3	7,00	104	F	1	0	4.5	Gym	7
DMB	4,00	35	F	1	0	0	Rhythmic gymnastics	8
DNC	10,00	3	F	1	0	NA	NA	NA
DR	4,00	4	M	1	0	NA	NA	NA

EKR	4,00	3	F	1	0	NA	NA	NA
FF2	11,00	115	F	1	0	0	No sport	3
JSR	12,00	87	M	1	0	NA	NA	NA
LGA	8,00	148	M	1	0	0	Calisthenics	8
LMF	2,00	107	M	1	0	NA	NA	NA
LF	9,00	114	M	1	0	0	Gym	6
MR	10,00	3	M	1	0	NA	NA	NA
MM2	11,00	41	F	1	0	0	Swimming	6
MMC	11,00	60	F	1	0	0	Swimming	6
MG	3,00	95	M	1	0	0	Basketball	8
MIP	9,00	45	F	1	0	NA	NA	NA
MG	5,00	37	M	1	0	0	No sport	3
NV	12,00	92	F	1	0	0	Gym	7
NM	9,00	3	F	1	0	NA	NA	NA
PC	13,00	88	F	1	0	0	Figure skating	8
PAA	9,00	104	M	1	0	0	Athletics	8
PS	5,00	49	M	1	0	NA	NA	NA
RL	13,00	52	F	1	0	NA	NA	NA
RP	8,00	121	F	1	0	0	No sport	3
RG	7,00	100	M	1	0	0	Soccer	8
RN	6,00	91	M	1	0	0	Soccer	8
RT	10,00	3	F	1	0	NA	NA	NA
SA	13,00	88	M	1	shoulder instability	0	Gym	7
SC	9,00	37	F	1	0	NA	NA	NA
SA2	2,00	1	F	1	0	NA	NA	NA
SF	12,00	85	M	1	0	0	Gym	7
SB	13,00	40	F	1	0	NA	NA	NA
TZ	4,00	3	F	1	0	NA	NA	NA
TL	1,00	88	F	1	skin irritation	0	Swimming	6
TL3	8,00	84	M	1	0	0	Basketball	8
TM2	11,00	99	M	1	0	0	Boxing	8
TI	4,00	3	M	1	0	NA	NA	NA

UM	11,00	3	M	1	0	NA	NA	NA
WZX	3,00	56	F	1	0	0	No sport	3
WYY	8,00	44	M	1	0	0	NA	NA
ZBD	6,00	96	M	1	0	0	Volleyball / running	8
ZB	12,00	95	M	1	0	0	Soccer	8
AEA	6,00	3	F	2	0	NA	NA	NA
AA2	10,00	75	F	2	0	0	Artistic gymnastics	8
BE	12,00	100	M	2	0	0	No sport	3
BM	5,00	3	F	2	0	NA	NA	NA
BF	12,00	137	M	2	0	0	Gym	7
BD	13,00	61	M	2	0	0	Soccer	8
BG3	9,00	3	F	2	0	NA	NA	NA
BC	8,00	183	M	2	0	0	gym	7
BG4	9,00	99	F	2	0	0	No sport	3
BC2	11,00	36	M	2	0	0	Basketball	8
BM2	12,00	93	M	2	0	0	Volleyball	8
CA	1,00	3	F	2	0	NA	NA	NA
CC	10,00	60	F	2	0	0	volleyball	8
CA2	11,00	3	F	2	0	NA	NA	NA
CG2	8,00	3	M	2	0	NA	NA	NA
DPE	13,00	50	M	2	0	0	Swimming	6
DGM	12,00	89	M	2	0	0	Soccer	8
DPG	10,00	158	M	2	0	0	play drum	5
EM	13,00	70	M	2	0	9	No sport	3
FF	6,00	51	M	2	0	0	soccer	8
FA	9,00	62	F	2	0	0	Horse riding	6
GC	12,00	52	F	2	0	0	gym	7
GMA	6,00	85	F	2	0	0	No sport	3
HLH	10,00	31	M	2	0	NA	NA	NA
LCFF	8,00	48	M	2	0	0	water polo	8
MF	8,00	90	M	2	0	0	No sport	3

ML	12,00	3	M	2	0	NA	NA	NA
MC	10,00	97	M	2	0	0	Soccer	8
MA	4,00	3	M	2	0	NA	NA	NA
MM3	4,00	59	M	2	0	0	judo	8
MS	7,00	39	F	2	0	0	volleyball	8
MS1	12,00	44	M	2	0	0	motocross	9
MG	8,00	38	M	2	0	0	Soccer	8
MG1	11,00	63	F	2	0	0	volleyball	8
ND	10,00	43	M	2	0	0	Basketball	8
PM	12,00	107	M	2	0	NA	NA	NA
PF	11,00	112	M	2	0	0	gym	7
PS	12,00	93	F	2	0	0	gym	7
PE	13,00	117	M	2	0	0	gym	7
PL	7,00	71	F	2	0	0	volleyball	8
PMC	12,00	38	F	2	0	0	No sport	3
RH	10,00	41	F	2	0	0	tennis	8
RM	8,00	27	M	2	0	NA	NA	NA
RS	9,00	67	F	2	0	0	Figure skating	8
TG	9,00	67	M	2	0	2.3	Basketball	8
TL2	7,00	96	F	2	0	0	Figure skating	8
TS	10,00	80	F	2	0	0	swimming/volleyball	7
TP	12,00	95	M	2	0	0	basketball/cycling	8
TS2	10,00	34	F	2	0	0	tennis	8
TR	12,00	180	M	2	0	0	gym	7
VMA	10,00	50	M	2	0	0	basketball	8
ZMG	10,00	80	F	2	0	0	Athletics	8
EYH	12,00	3	F	2	0	NA	NA	NA
CE2	10,00	88	F	3	0	0	Volleyball/dance	8
CC2	5,00	3	F	3	0	NA	NA	NA
DR	10,00	59	M	3	0	0	water polo	7
FM	11,00	53	F	3	0	0	tennis	8
FM2	10,00	180	M	3	0	0	gym	6

GA	12,00	3	M	3	0	NA	NA	NA
MG	7,00	3	F	3	0	NA	NA	NA
MK	7,00	45	F	3	0	0	Athletics	8
MF	5,00	4	F	3	0	NA	NA	NA
MC	12,00	45	F	3	0	0	Artistic gymnastics	8
PG	9,00	57	F	3	0	0	karate	8
RM	9,00	96	F	3	0	0	Artistic gymnastics	8
SB	7,00	3	F	3	0	NA	NA	NA
SBM	7,00	36	F	3	0	0	judo	8
TM	11,00	39	M	3	0	0	Boxing	8
VE	4,00	49	M	3	0	0	swimming	6
FD	6,00	13	M	4	0	0	swimming	6
LA	7,00	3	M	4	0	NA	NA	NA
